# Contribution of Estrone Sulfate to Cell Proliferation in Aromatase Inhibitor (AI) -Resistant, Hormone Receptor-Positive Breast Cancer

**DOI:** 10.1371/journal.pone.0155844

**Published:** 2016-05-26

**Authors:** Toru Higuchi, Megumi Endo, Toru Hanamura, Tatsuyuki Gohno, Toshifumi Niwa, Yuri Yamaguchi, Jun Horiguchi, Shin-ichi Hayashi

**Affiliations:** 1 Department of Molecular and Functional Dynamics, Graduate School of Medicine, Tohoku University, Sendai, Miyagi, Japan; 2 Department of Visceral and Thoracic Organ Surgery, Graduated School of Medicine, Gunma University, Maebashi, Gunma, Japan; 3 Division of Breast and Endocrine Surgery, Department of Surgery, Shinshu University School of Medicine, Matsumoto, Nagano, Japan; 4 Research Institute for Clinical Oncology, Saitama Cancer Center, Ina, Saitama, Japan; 5 Center for Regulatory Epi genome and Diseases, Graduate School of Medicine, Tohoku University, Sendai, Niyagi, Japan; University of South Alabama Mitchell Cancer Institute, UNITED STATES

## Abstract

Aromatase inhibitors (AIs) effectively treat hormone receptor-positive postmenopausal breast cancer, but some patients do not respond to treatment or experience recurrence. Mechanisms of AI resistance include ligand-independent activation of the estrogen receptor (ER) and signaling via other growth factor receptors; however, these do not account for all forms of resistance. Here we present an alternative mechanism of AI resistance. We ectopically expressed aromatase in MCF-7 cells expressing green fluorescent protein as an index of ER activity. Aromatase-overexpressing MCF-7 cells were cultured in estrogen-depleted medium supplemented with testosterone and the AI, letrozole, to establish letrozole-resistant (LR) cell lines. Compared with parental cells, LR cells had higher mRNA levels of steroid sulfatase (STS), which converts estrone sulfate (E1S) to estrone, and the organic anion transporter peptides (OATPs), which mediate the uptake of E1S into cells. LR cells proliferated more in E1S-supplemented medium than did parental cells, and LR proliferation was effectively inhibited by an STS inhibitor in combination with letrozole and by ER-targeting drugs. Analysis of ER-positive primary breast cancer tissues showed a significant correlation between the increases in the mRNA levels of STS and the OATPs in the LR cell lines, which supports the validity of this AI-resistant model. This is the first study to demonstrate the contribution of STS and OATPs in E1S metabolism to the proliferation of AI-resistant breast cancer cells. We suggest that E1S metabolism represents a new target in AI-resistant breast cancer treatment.

## Introduction

Aromatase inhibitors (AIs) block estrogen production from androgens and are routinely administered to postmenopausal women with estrogen receptor (ER)-positive breast cancer. AI efficacy was validated by several clinical trials [1–3], but some patients do not respond to this treatment and experience recurrence. Although not high, the rate of recurrence has remained almost constant for the first several years after treatment initiation [1]. The mechanisms of AI resistance in ER-positive postmenopausal breast cancer are incompletely identified. Thus far, they include ligand-independent activation of ERs [4–7] and signaling via human epidermal growth factor receptor 2 (HER2) [8, 9]. Mechanistic studies have identified mammalian target of rapamycin as a molecular target in AI resistance [10]; an inhibitor of this molecule was developed and a clinical study supports its efficacy [11]. Exploring genes of tissues from neoadjuvant clinical trials and patient-derived xenograft studies suggest that the somatic mutation of multiple genes and ESR1 mutation can also induce AI resistance [12, 13].

Our previous study showed that ER-positive breast cancer cells simultaneously acquired multiple AI resistance properties, including ER-independent and ER-dependent proliferation, when cultured in estrogen-depleted medium [14]. We also previously established two AI-resistant, androgen-dependent cell lines by culturing ER-positive cells in estrogen-depleted, androgen-supplemented medium [15, 16]; this condition mimics the microenvironment of AI-treated tumors (estrogen-depleted and androgen-enriched rather than simply estrogen-depleted) [17]. We suggest that different breast tumors have different AI resistance mechanisms, and a greater understanding of AI resistance is, therefore, essential.

Although decreases in ER activity and subsequent increases in HER2 activity (and consequent elicitation of proliferative signals) promote AI resistance [8, 9], ER expression does not change or only slightly decreases in recurrent tumors [18, 19]. Reports comparing ER expression in primary tissues and recurrent sites suggest that AI-resistant cell lines that continue to express ERs may be more realistic models of AI-resistant breast cancers. To establish a realistic model, we ectopically expressed aromatase in MCF-7-derived E10 cells, which express green fluorescent protein (GFP) as an index of ER activity [20]. We cultured aromatase-expressing cells in estrogen-depleted medium containing testosterone (TS) and the AI, letrozole (Let), and ultimately established Let-resistant (LR) cell lines.

Potential mechanisms that might account for the AI resistance of LR cells include not only dependence on testosterone metabolites or androgen receptors (ARs) [15, 16], but also enhanced catabolism and increased drug efflux [21–23]. Herein, we demonstrated that activation of steroid sulfatase (STS) and organic anion transporter peptides (OATPs) could make breast cancer cells acquire AI resistance. STS was previously reported as a mechanism for AI resistance [24], but this study is the first to report that the induction of OATP transporting estrone sulfate (E1S) in addition to STS caused AI resistance in breast cancer cell lines. An analysis of mRNA expression in ER-positive breast cancer tissues supports the clinical significance of this mechanism. The results of this study suggest an alternative endocrine therapy against AI-resistant breast cancer.

## Materials and Methods

### Steroid hormones and reagents

Testosterone (TS), estradiol (E2), estrone (E1), estrone sulfate (E1S), STX64, and 4-OH tamoxifen were purchased from Sigma-Aldrich (St. Louis, MO, USA). Letrozole (Let) was kindly provided by Novartis Pharmaceuticals (Basel, Switzerland) and ICI 182780 (fulvestrant) by Astra Zeneca K.K. (Osaka, Japan).

### Cell culture, plasmids, and transfections

MCF-7 and previously established E10 were authenticated by STR analysis to be the same as the cells registered in the cell banks such as JCRB, DSMZ and ATCC [14, 15, 20]. Cells were cultured in phenol red-free RPMI 1640 medium (referred to as medium hereafter; GIBCO BRL, Grand Island, NY, USA) supplemented with 5% or 10% dextran-coated, charcoal-treated (DCC) fetal calf serum (FCS) (Tissue Culture Biologicals, Tulare, CA, USA) and 1% penicillin/streptomycin. An aromatase-overexpressing cell line (E10arom) was established by inserting the genes encoding aromatase and blasticidin into pRL-CMV plasmids lacking the Renilla luciferase gene; the resulting plasmid was stably transfected into E10 cells using TransIT LT-1 transfection reagent (TaKaRa Bio Inc., Otsu, Japan) according to the manufacturer’s protocol. To establish cell lines overexpressing steroid sulfatase (STS, cell line termed MCF-STS), the human STS gene in pRC/CMV was stably expressed in MCF-7 cells as described above. E10arom was established from single clone. As positive control in measuring aromatase mRNA, stromal cells previously established were used [25], E10arom constitutively expressed aromatase gene (S1A Fig). During repeated passages, E10arom was cultivated in steroid-depleted medium supplemented with BSD, and finally established as aromatase-overexpressing cell. Proliferation of this cell line was inhibited in Let-dependent manner (S1B Fig). The generation of the LR cell lines is described in the results section.

For transfection of siRNA, cells (5 × 10^4^) were cultured in medium containing 10% DCC-FCS for three days and transferred to 24-well plates in fresh medium containing 10% DCC-FCS. Twenty-four hours later, the cells were refed with medium (1 mL/well) containing 10 nM OATP4A1 siRNA (ID 2108, Sigma-Aldrich) or scrambled RNA (100 μL) and siLentFect Lipid Reagent for siRNA (2.5 μL; Bio-Rad, Hercules, CA, USA). Cells were incubated for 48 h before harvest.

### Reverse transcription PCR (RT-PCR) and real-time PCR

Cells were grown to approximately 70% confluence in 6-cm dishes, and total RNA was extracted with acid guanidinium phenol-chloroform containing ISOGEN (Nippon Gene, Toyama, Japan) as a protein denaturant according to the manufacturer’s protocol. cDNA was produced from 1 μg RNA using the Quantitect RT-PCR kit (Qiagen, Mississauga, Ontario, Canada). mRNA levels were measured using a StepOne Real-Time PCR system (Applied Biosystems Inc., Foster City, CA, USA). The PCR reactions (20 μL total volume) contained 10 μL Brilliant III Ultra-Fast SYBR Green QPCR Master Mix and 2 μL cDNA. The concentration of the reference dye and primers was 15 nM. Primer sequences are shown in S1 Table, and the real-time PCR protocol is outlined in S2A Table. mRNA expression of AR, AR target genes, androgen-metabolizing enzymes, and drug efflux transporters were analyzed in duplicate in triplicate plates. All other mRNA expressions were analyzed in triplicate in triplicate plates.

For determination of mRNA expression in clinical samples, real-time PCR was performed using TaqMan Fast Universal PCR Master Mix and TaqMan primers and probe sets (Applied Biosystems) as specified by the manufacturer. This protocol and the IDs of the primer and probe sets are shown in S2B and S2C Table. The amount of mRNA in cultured cells and clinical samples was normalized to the amount of RPL13A and β-actin, respectively.

### Luciferase assay

Cells were cultured in steroid-depleted medium for three days and seeded in 6-cm dishes (10,000 cells/dish). Twenty-four hours after seeding, cells were transfected with a plasmid encoding a luciferase reporter driven by the estrogen response element or pRL-tk-luciferase in medium containing TransIT LT-1 transfection reagent (TaKaRa). Forty-eight hours after transfection, luciferase activity was measured in triplicate using a luciferase assay system (Promega, Madison, WI, USA).

### Proliferation assay

LR and parental E10 cells were cultured for three days in medium containing 10% DCC-FCS and transferred to 24-well plates (10,000 cells/well) in medium containing 10% DCC-FCS and the test compounds, except for E1S. For E1S assays, LR and parental cells were cultured in 24-well plates (10,000 cells/well) in medium containing 10% DCC-FBS for three days and refed with medium containing 10% DCC-FCS and E1S. MCF-STS cells were cultured in medium containing 5% DCC-FCS for three days and transferred to 24-well plates (10,000 cells/well) in medium containing 5% DCC-FCS and E1S. Cells were harvested 4 days (LR and parental) or 5 days (MCF-STS) after addition of the test compounds and were counted using a micro cell counter (Sysmex, Hyogo, Japan). Assays were carried out in triplicate.

### Measurement of E1S concentration via enzyme immunoassay

E1S levels in the culture medium were measured in triplicate using the DetectX estrone-3-sulfate enzyme immunoassay kit (Arbor Assays, Ann Arbor, MI, USA). Briefly, cells in 24-well plates (10,000 cells/well) were incubated for three days in medium containing 10% DCC-FCS and refed with medium containing 10% DCC-FCS and 1 μM E1S. Media was harvested four days after refeeding, centrifuged, and the supernatant was diluted 1:100 in medium before assaying.

### Clinical tissue samples

This research was conducted according to the Declaration of Helsinki Principles. Under written informed consent, all clinical tissues were resected from patients who underwent breast surgery at Gunma University Hospital from May 2011 to May 2012. Analysis of mRNA expression in clinical tissues was approved by the Gunma University Hospital Ethics Committee. All tissues were immediately treated with RNAlater (Sigma-Aldrich). Total mRNA was extracted using RNeasy and a mini kit (Qiagen).

### Statistical analysis

All data is presented as the mean ± standard deviation, and differences between means were tested for significance. Correlations between mRNA expression and specific parameters were assessed using the Pearson correlation test with 5% significance; for simplicity, mRNA expression values were converted to base 2 logarithms. Statistical analysis was performed using SPSS version 21 software (IBM, Armonk, NY, USA).

## Results

### Establishment of an ER-expressing, AI-resistant model from E10arom cells

**Fig 1:**
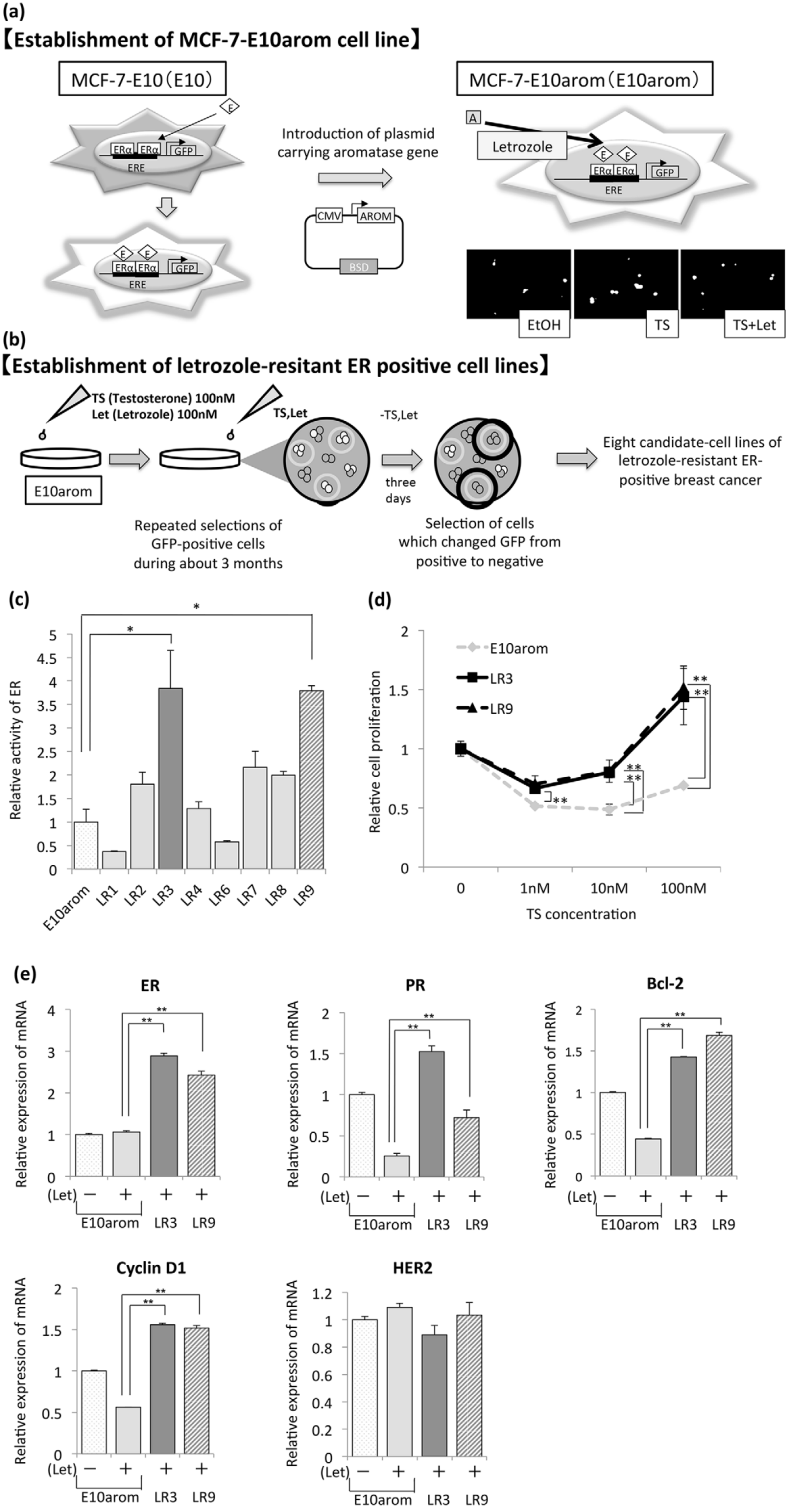
Establishment of letrozole-resistant (LR) cell lines. **A)** Schematic representation of the establishment of E10arom cells from E10 cells. Estrogen receptor (ER) activity was visually monitored in cells treated with testosterone (TS) or TS plus letrozole (Let) via green fluorescent protein (GFP) expression. The green color indicates GFP expression. Comparison of the three images shows that E10arom cells express more GFP when treated with TS alone than with TS plus Let or when untreated (control). E estrogen, A androgen, CMV cytomegalovirus promoter, AROM aromatase gene, BSD blasticidin gene, ERE estrogen response element, EtOH ethanol. **B)** Eight candidate ER-expressing, Let-resistant cell lines. These cell lines are circled in black. **C)** ER activity in the LR cell lines was measured using a luciferase reporter driven by the estrogen response element. **D)** Cell number was determined in LR cultures receiving 100 nM Let and the indicated concentrations of TS. The vertical axis indicates cell number relative to vehicle-treated controls. **E)** mRNA expression of ERs, ER target genes including cyclin D1, bcl-2 and progesterone receptor (PR), and human epidermal growth factor receptor 2 (HER2) was determined via real-time PCR. Values are normalized to that of the RPL13A gene. Error bars indicate standard deviation. * p < 0.05. ** p < 0.01.

ER-expressing, AI-resistant cells were established as follows. First, a plasmid carrying the aromatase gene was stably introduced into E10 cells (E10arom cells; Fig 1A, left diagram) [20]. As monitored by GFP expression, ER activity increased in TS-treated E10arom cells and decreased in cells co-treated with TS and Let compared with ethanol-treated control cells (Fig 1A, fluorescent images). Second, E10arom cells were cultured in estrogen-depleted medium supplemented with 100 nM TS and 100 nM Let for three months (Fig 1B). ER activity was monitored every time cells were passaged to distinguish these cells from AI-resistant cells with reduced ER activity that proliferate in response to signals generated by other growth factors [8, 9]. Ultimately, eight candidate cell lines (termed “LR” for “letrozole-resistant”) were selected (Fig 1B); the two with the significantly highest ER activity without E2 (LR3 and LR9) compared with control were further studied (Fig 1C).

LR3 and LR9 cells grew better than E10arom cells in estrogen-depleted medium supplemented with TS and Let (Fig 1D) and, therefore, were considered Let-resistant. They also had significantly higher mRNA levels of ER and ER target genes including progesterone receptor (PR), bcl-2, and cyclin D1, whereas mRNA levels of HER2 were similar in Let-resistant and E10arom cells (Fig 1Ee). These findings suggest that LR cells acquire Let resistance while maintaining their ER activity and without inducing HER2 signaling.

### Investigation of the hypothesized mechanism of AI resistance in LR cell lines

Referring to previous reports [15, 16, 26], we hypothesized that LR proliferation required androgens and ARs because of the abundance of TS in the culture medium. LR3 and LR9 cells expressed different amounts of AR mRNA and, compared with parental E10arom cells, dramatically reduced amounts of the mRNA encoding the AR target gene, kallikrein-related peptidase 3 (S2A Fig). We asked whether androgens might function as ER ligands [15]. mRNA expression of androgen-metabolizing enzymes (3-β hydroxyl steroid dehydrogenase type 1, aldo-keto reductase 1C3, and steroid 5α-reductase type 1) differed between the LR cell lines (S2A Fig), suggesting that LR cell lines did not acquire AI resistance via induction of androgen-metabolizing enzymes or enhanced use of androgen metabolites.

As noted above, drug-resistant mechanisms often involve the activation of catabolic enzymes and enhanced drug efflux. Therefore, we monitored the mRNA expression of breast cancer-resistant protein (BCRP) and the ATP-binding cassette transporter B1 (ABCB1) [21, 22, 23]. Expression of cytochrome p450 3A4 (CYP3A4) [27], transcription factors for nuclear receptors, and constitutive androstane and pregnane X receptor [28] were also monitored, although their involvement, except CYP3A4, in AI catabolism has not been demonstrated. Compared with HepG2 cells, which abundantly express these metabolic enzymes, and thus, are a suitable positive control, none of these mRNAs was appreciably expressed in LR cells (S2B Fig). These observations suggest that LR cell lines do not require any of the above-mentioned proteins for AI resistance.

### Estrogen production as a novel mechanism of AI resistance in LR cell lines

**Fig 2:**
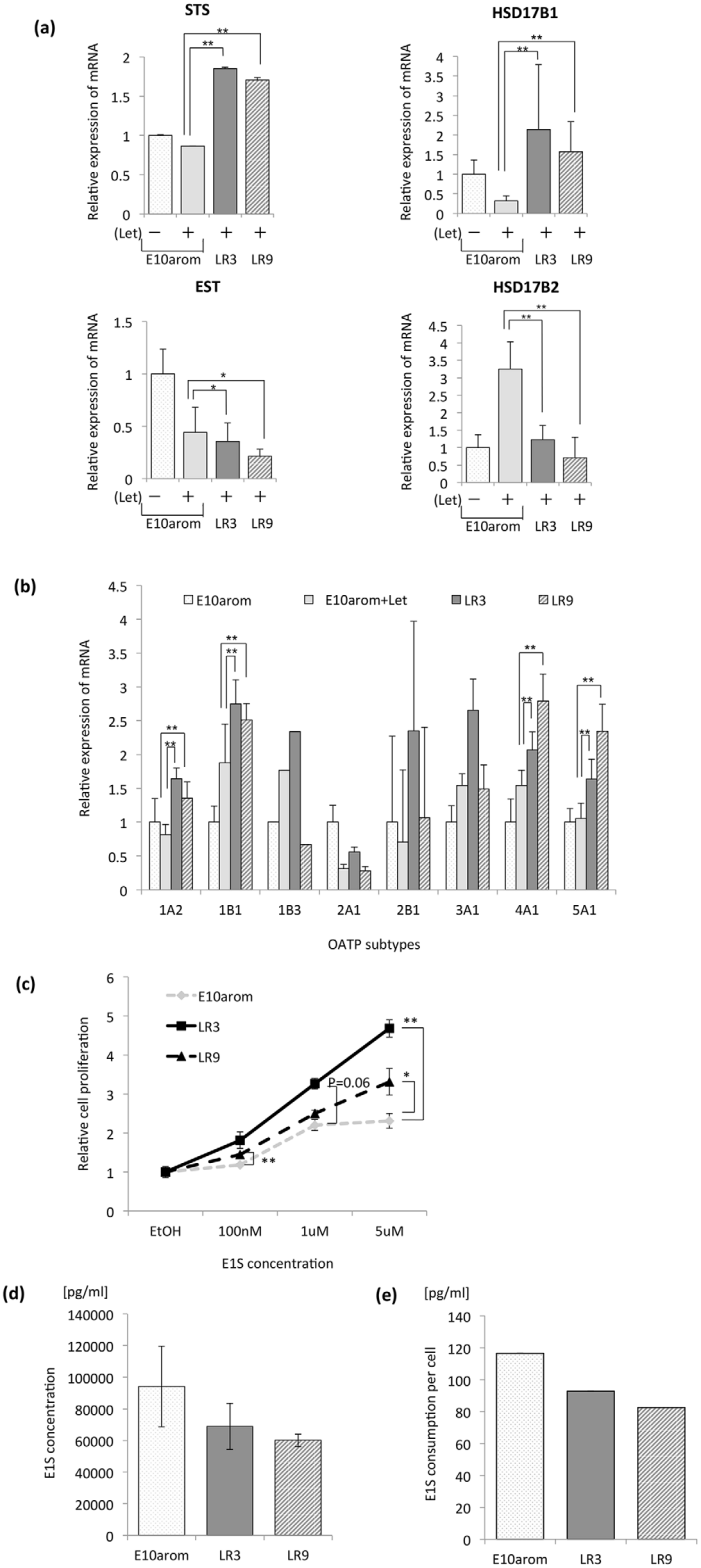
The contribution of estrone sulfate (E1S) to the proliferation of letrozole (Let)-resistant (LR) cells. **A)** Relative mRNA expression of steroid sulfatase (STS) in LR cell lines and parental cells. Let letrozole, HSD17B1 17β hydroxyl steroid dehydrogenase type 1, EST estrone sulfotransferase, HSD17B2 17β hydroxyl steroid dehydrogenase type 2. **B)** Expression of organic anion transporter peptides (OATPs) mRNA. In Fig 2A and 2B, values are normalized to that of the RPL13A gene. **C)** Proliferation of LR and E10arom cell lines treated with E1S. **D)** E1S consumption by LR cells in E1S-containing medium measured via enzyme immunoassay. **E)** E1S consumption per cell. Consumption was measured by dividing the number of E1S molecules (determined via enzyme immunoassay) by the number of cells (obtained in proliferation assays of cells receiving 1μM E1S). Error bars show standard deviation. * p < 0.05. ** p < 0.01.

Estrogen is produced not only by aromatization of androgens but also by the conversion of E1S to E1 by STS [29–32]. E1S is the most abundant estrogenic hormone in the plasma of postmenopausal woman [33, 34], and we, therefore, considered enhanced E1S metabolism as a possible mechanism of AI resistance in our model. To address this possibility, we monitored the mRNA expression of STS and OATPs, which transport E1S into cells [35–38]. STS mRNA levels were significantly higher in LR3 and LR9 cells than parental E10arom cells, as were those of the four OATPs (1A2, 1B1, 4A1, and 5A1) (Fig 2A and 2B). mRNA levels of 17β hydroxyl steroid dehydrogenase type 1 (HSD17B1), which converts E1 to estradiol (E2), were also significantly higher in the LR cell lines. In contrast, mRNA levels of estrone sulfotransferase (which converts E1 to E1S) and 17β hydroxyl steroid dehydrogenase type 2 (which converts E2 to E1) were lower (Fig 2A).

Treatment of LR cells with E1S significantly increased proliferation in a dose-dependent manner compared with E10arom cells (Fig 2C), while treatment with E1 or E2 did not (S3 Fig). To compare E1S consumption in LR and E10arom cells, we measured the amount of residual E1S in E1S-supplemented culture media (Fig 2D). E1S levels were lower in the LR medium, but not significantly so. To determine the consumption of E1S per cell, the number of remaining E1S molecules in the medium (Fig 2d) was divided by cell number (Fig 2E). The results of this calculation showed a tendency toward greater E1S consumption by the LR cells than the parental E10arom cells. Collectively, our data suggests that LR cells rely more heavily on E1S for proliferation than do E10arom cells.

### Inhibition of LR proliferation by STX64 plus Let and ER inhibitors

**Fig 3:**
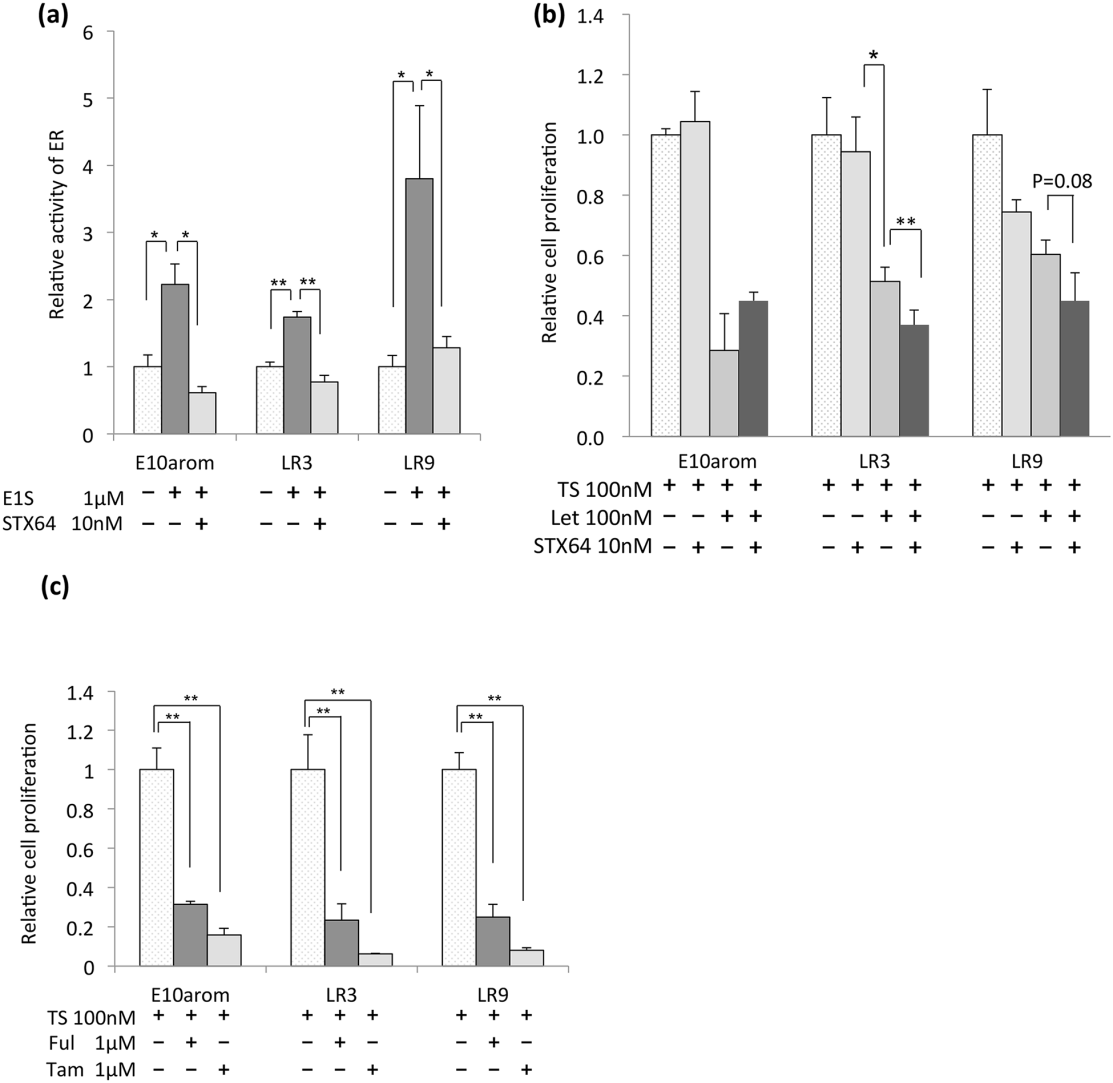
Effects of a steroid sulfatase (STS) inhibitor and estrogen receptor (ER) inhibitors on ER activity and proliferation in letrozole (Let)-resistant (LR) cell lines. **A)** Relative ER activity in LR cell lines and parental cells treated with or without the STS inhibitor STX64. **B)** The relative proliferation of LR and parental cells treated with testosterone (TS) in the presence or absence of Let, STX64 or both. **C)** Relative cell proliferation with or without 4-OH tamoxifen (tam) and fulvestrant (ful). Error bars show standard deviation. * p < 0.05. ** p < 0.01.

To confirm the E1S-dependent cell growth in our AI-resistant model, we investigated whether the STS inhibitor, STX64, affected the ER activity and growth of LR cells. E1S increased ER activity in both LR cell lines in the absence, but not presence, of STX64 (Fig 3A), and E1 and E2 rescued LR cells from the inhibitory effects of STX64 (S4 Fig). LR cell lines were more inhibited by STX64 than E10arom, while they were less inhibited by Let than the parental E10arom (Fig 3B). Compared to treatment of only STX64, STX64 plus Let significantly or marginally significantly inhibited the proliferation of LR cells, though only LR3 was significantly inhibited by STX64. An estrogen receptor modulator (SERM) and an estrogen receptor down-regulator (SERD) (the ER inhibitors tamoxifen and fulvestrant, respectively), also effectively inhibited the proliferation of LR and E10arom cells treated with TS (Fig 3C), suggesting that LR cells were more dependent on the STS-OATP pathway for estrogen production than E10arom cells.

### E1S-dependent cell growth in MCF-7 cells overexpressing STS

To further examine the role of E1S and STS in ER-positive breast cancer, we established a cell line that overexpressed STS (MCF-STS) (S5A Fig). MCF-STS cells proliferated to a significantly higher extent than did parental MCF-7 cells in the presence of EIS (S5B Fig), suggesting that STS and E1S promote the proliferation of ER-positive breast cancer cells.

### Clinical significance of E1S-dependent growth in AI resistance

**Fig 4:**
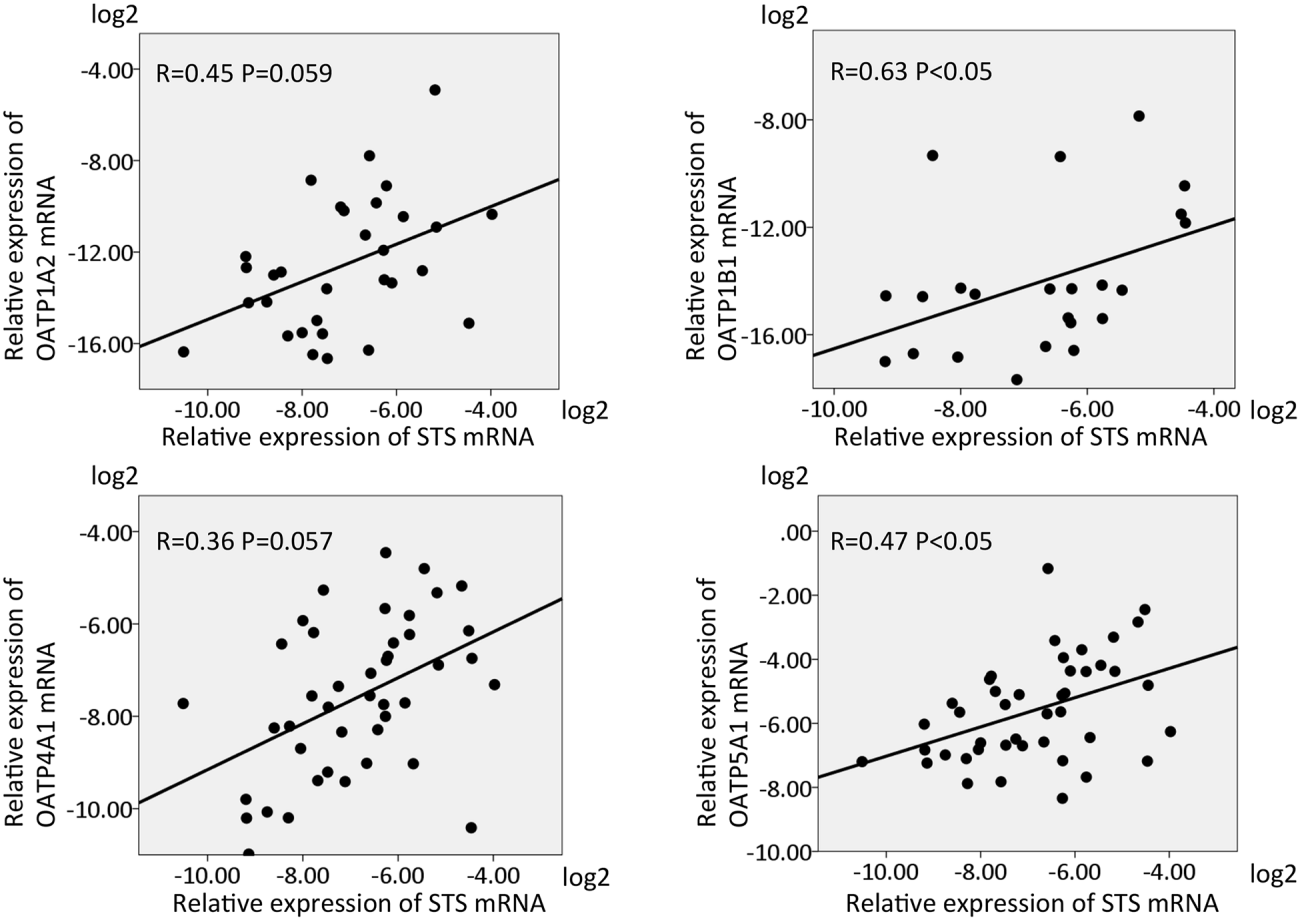
Comparison of steroid sulfatase (STS) mRNA and organic anion transporter peptides (OATPs) mRNA expression in primary breast cancer tissues. Values were normalized to that of β-actin and converted to base 2 logarithms. R indicates the correlation index.

We measured the mRNA levels of STS and OATPs in primary breast cancer tissue samples of postmenopausal patients to assess the clinical significance of our model. STS mRNA levels were significantly or marginally significantly correlated with those of the four OATPs upregulated in the LR cell lines (Fig 4).

## Discussion

**Fig 5:**
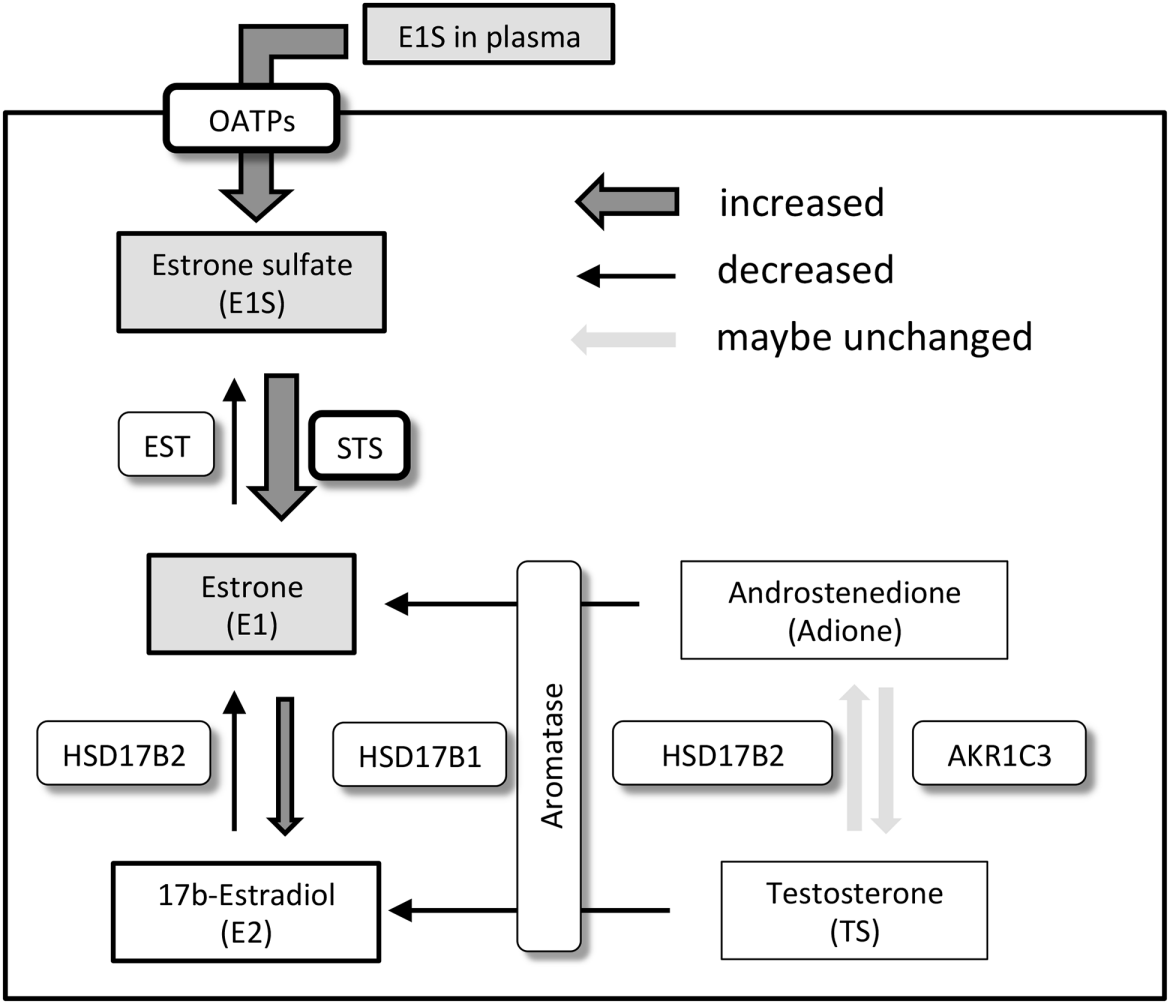
Putative model of steroid metabolism in letrozole-resistant (LR) cell lines. The map shows our model of steroid metabolism in LR cell lines. We suggest that LR cell lines acquire resistance to aromatase inhibitors via augmentation of STS activity. EST estrone sulfotransferase, STS steroid sulfatase, HSD17B 17β hydroxyl steroid dehydrogenase, HSD3B1 3β hydroxyl steroid dehydrogenase type 1, SRD5A1 steroid 5α-reductase type 1, AKR1C3 aldo-keto reductase 1C3. HSD17B1 1, 2, and 5 refer to three different forms of this enzyme.

AIs are widely used to prevent the recurrence of ER-positive postmenopausal breast cancers. However, some patients are resistant to AIs, and the mechanism of resistance is incompletely understood. Via establishment of LR cell lines, we show that E1S contributes to the growth of ER-positive, AI-resistant breast cancer cells. STS plus Let effectively inhibited the growth of LR cells, as did SERM and SERD. As diagrammed in Fig 5, our results suggest that LR cells produce estrogens from E1S rather than androgens by inducing the expression of STS and OATPs.

Several reports suggest that high STS mRNA levels correlate with poor prognosis [39, 40]. Three STS inhibitors have been developed [41–43] and are currently being tested in clinical trials [41, 43]. Meanwhile, whether OATP inhibitors (which have yet to be developed) would be clinically useful is still unclear. Several reports have localized OATPs within the cell via immunohistochemistry or immunofluorescence [35–38, 44–46]; thus, targeting of OATP inhibitors to OATPs can be achieved. However, there are at least ten OATP subtypes [37, 47], and the role of OATP in the breast or other organs has yet to be identified. In our silencing experiment, depletion of the most highly induced OATP in LR cells, OATP4A1, did not alter the E1S dose-dependent cell proliferation (S6 Fig), suggesting that loss of a single OATP is insufficient for restriction of E1S influx and consequent AI resistance. Notably, this study is the first to suggest that OATPs acting as E1S transporters might be involved in the AI resistance of ER-positive breast cancer. Validation of the role of OATPs in AI resistance requires further investigation.

HSD17B1 is an enzyme important in estrogen production in postmenopausal women [48], but its role in E1S-dependent, ER-positive breast cancer is unclear. The growth of LR cells in E1-supplemented medium was similar to that of parental cells in E1-supplemented medium, as was ER activity (S4 Fig), suggesting that E1 usage is also similar. ERs have a 50% lower affinity for E1 than E2 [49], which may explain how LR cells acquired AI resistance (i.e., by converting E1S to E1). HSD17B1 expression is not related to prognosis in ER-positive breast cancer, [40] and is similar in ER-positive and ER-negative breast cancers [38]. Therefore, HSD17B1 may not be an effective target in E1S-dependent, ER-positive, AI-resistant breast cancer treatment.

Treatment after recurrence is generally based on the immunohistological status of ERs, PRs, and HER2s in primary breast cancer; however, the expression of these markers is not synonymous with recurrence. Consequently, treatment efficacy in recurrent cases cannot be assessed in a short period of time. New biomarkers of recurrence are always needed. Our results suggest that STS and OATP mRNAs may be novel indicators of the need for SERM or SERD treatment of recurrent AI-resistant breast cancers. As shown in our study, these agents inhibited the growth of LR cells. Therefore, if STS and OATP mRNAs are overexpressed in primary breast cancers or recurrent sites, SERM or SERD may effectively treat resistant and recurrent lesions. Needless to say, additional study of STS and OATP in the context of AI-resistant, ER-positive breast cancers is needed; however, their potential clinical significance should not be overlooked.

The concentration of E1S in estrogen-depleted medium was below the limit of detection in our study (data not shown). Because there are multiple signals for the survival of breast cancer cells [5, 9, 50, 51], the survival of LR cells might not completely depend on E1S. E1 and E2 have a much higher binding capacity for ERs than E1S [49], and ERs can become hypersensitive to estrogen in estrogen-limiting conditions [4]. In such conditions, LR cells could survive by producing active estrogen from small amounts of E1S in the medium and effectively utilizing estrogen. Alternatively, they may activate the STS-OATP pathway to produce estrogen. Low concentrations of E1S in the medium do not necessarily refute this mechanism of AI resistance.

This model demonstrated that dual-blockade of aromatase and steroid sulfatase effectively inhibited cell growth of AI-resistant breast cancer cell. Concurrent treatment of AI and endocrine-targeted agents are tested, but the ATAC study showed that concurrent treatment with non-steroidal AI and SERM did not significantly restrict recurrence more than the use of non-steroidal AI or SERM alone [1]. However, the combined therapy of endocrine-targeted drugs is not necessary invalid. LHRH agonist and SERM or AI treatment of some premenopausal breast cancer patients showed its efficacy through adjuvant phase III trials [52, 53]. The combination of treatments targeted towards endocrine pathways is a hopeful treatment for ER-positive breast cancer. Moreover, Baselga et al. showed that treatment of exemestane plus the molecular-target drug, mTOR inhibitor, is very effective for the improvement of progression-free survival of advanced breast cancer after the failure of non-steroidal AI treatment [11]. This study demonstrated the efficacy of AI with mTOR inhibitor, suggesting that concurrent treatment with multiple drugs including AI was hopeful for AI-resistant breast cancer. Thus far, combined treatments, including AI and STS, have not been validated by trials and are not clinically used. However, Rasmussen et al. reported that dual inhibition of STS and estrogen receptor effectively suppressed tumor growth [24], supporting the efficacy of concurrent treatments targeted to multiple endocrine pathways.

## Conclusion

In this study, we show that E1S contributes to AI resistance in breast cancer cells, and thus, identify alternative targets for overcoming resistance. It should be noted that our study is presumably the first to document that estrogen production from E1S was induced by activation of both STS and OATP in the context of AI resistance. Therapy targeting this metabolic pathway presents new options for the treatment of AI-resistant breast cancer, especially in combination with AIs.

## Supporting Information

S1 Fig Validation of the potentials of E10arom as aromatase-overexpression cell line. (PDF)

S2 Fig Searching for the proliferation driver in letrozole-resistant (LR) cells. (PDF)

S3 Fig Proliferation assay of LR cell lines and E10arom. (PDF)

S4 Fig Relative estrogen receptor (ER) activity assay. (PDF)

S5 Fig E1S-dependent cell growth in MCF-7 cells overexpressing STS. (PDF)

S6 Fig Effect of organic anion transporter peptide 4A1 (OATP4A1) siRNA on the proliferation of letrozole-resistant (LR) cells. (PDF)

S1 Table Primer sequences used in this study. (PDF)

S2 Table Real-time polymerase chain reaction protocols and IDs of the primers and probe sets. (PDF)
