# Supplementary material for: Contribution of Estrone Sulfate to Cell Proliferation in Aromatase Inhibitor (AI) -Resistant, Hormone Receptor-Positive Breast Cancer
Source: PLoS One. 2016 May 26;11(5):e0155844. doi: 10.1371/journal.pone.0155844 (PMC4882040; doi:10.1371/journal.pone.0155844)

**S1 Figure. Validation of the potentials of E10arom as aromatase-overexpression cell line. A)** RNA expression of aromatase of E10arom comparing to preciously established stromal cells and parental E10. **B)** Proliferation assay of E10arom with treatment of letrozole. DEX dexamethasone, TS testosterone, Let letrozole.

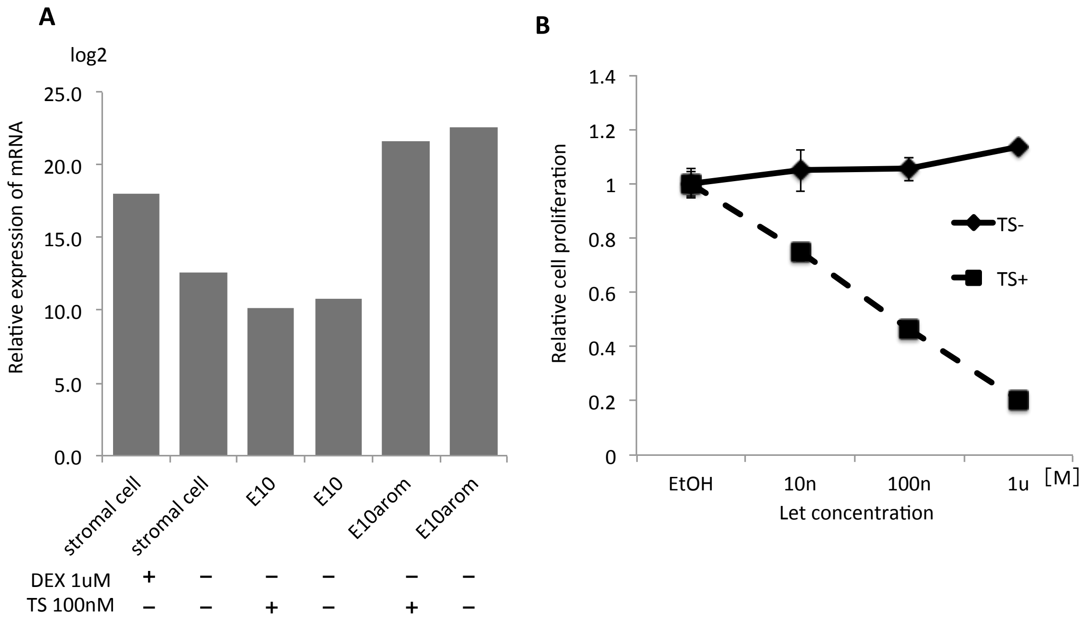

Supplement: S1 Fig — (PDF) [file pone.0155844.s001.pdf]
