# Supplementary material for: Contribution of Estrone Sulfate to Cell Proliferation in Aromatase Inhibitor (AI) -Resistant, Hormone Receptor-Positive Breast Cancer
Source: PLoS One. 2016 May 26;11(5):e0155844. doi: 10.1371/journal.pone.0155844 (PMC4882040; doi:10.1371/journal.pone.0155844)
Supplement: S2 Fig — (PDF) [file pone.0155844.s002.pdf]

S2 Figure. Searching for the proliferation driver in letrozole-resistant (LR) cells. A) Relative mRNA expression of the androgen receptor (AR), kallikrein-related peptidase 3 (KLK3, a product of an AR-targeted gene), and androgen-metabolizing enzymes. HSD3B1 3-β hydroxyl steroid dehydrogenase type 1, SRD5A1 steroid 5α-reductase type 1, AKR1C3 aldo-keto reductase 1C3. **B)** mRNA expression of drug transporters and aromatase-metabolizing enzymes. Error bars show standard deviation. BCRP breast cancer resistance protein, ABCB1 ATP-binding cassette subfamily B member 1, CYP3A4 cytochrome P450 monooxygenase 3A4, CAR constitutive androstane receptor, PXR pregnane X receptor.

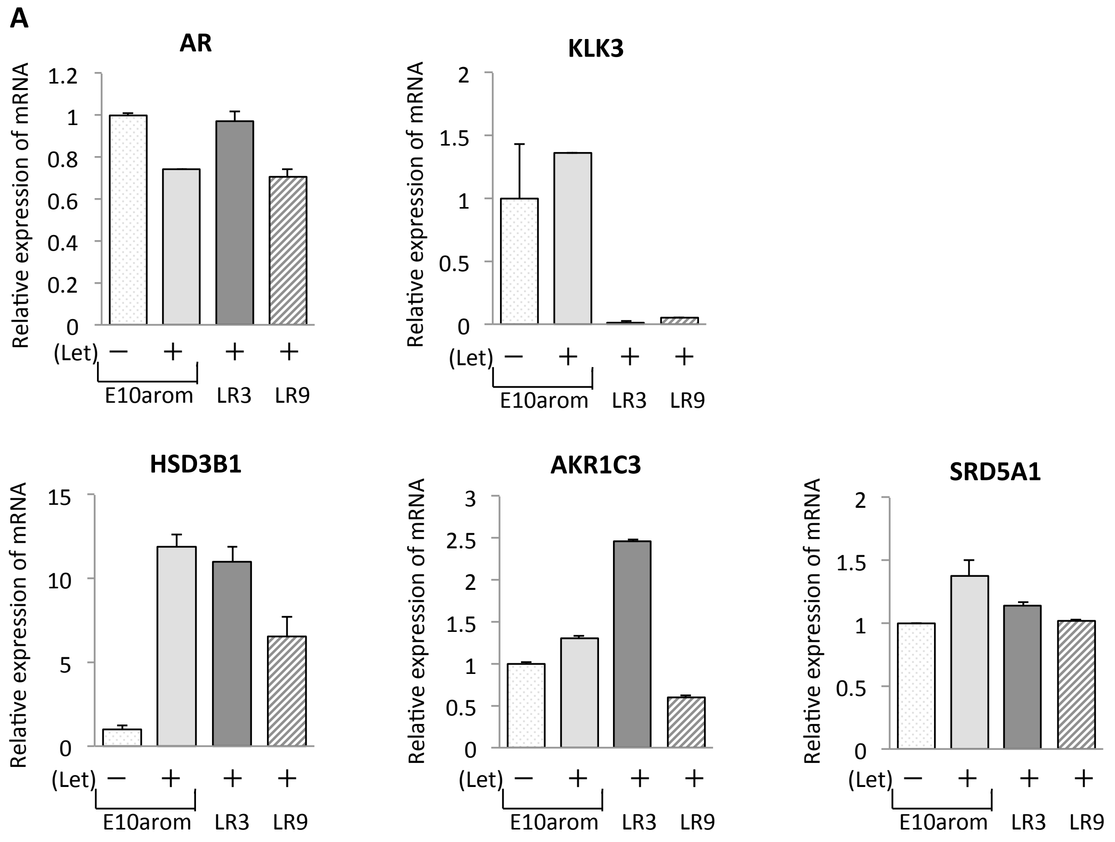

B

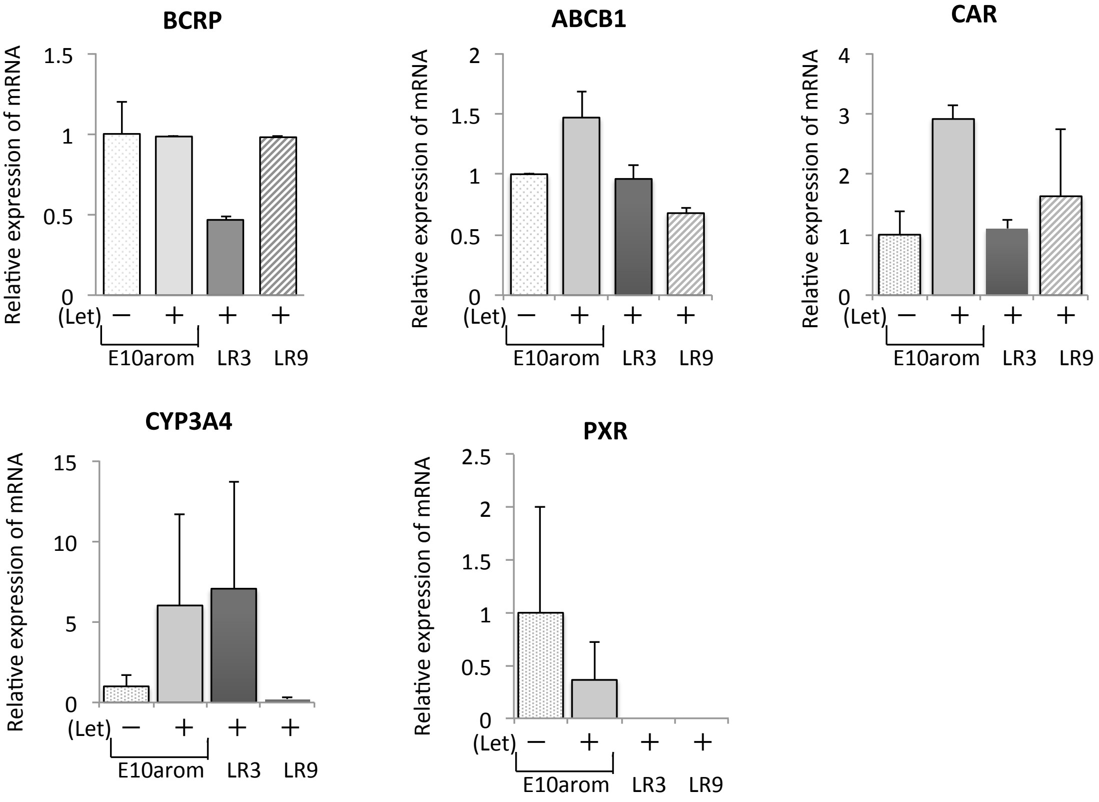
BCRP
Relative expression of mRNA
1.5
1
0.5
0
(Let) − + + +
E10arom LR3 LR9
ABCB1
Relative expression of mRNA
2
1.5
1
0.5
0
(Let) − + + +
E10arom LR3 LR9
CAR
Relative expression of mRNA
4
3
2
1
0
(Let) − + + +
E10arom LR3 LR9
CYP3A4
Relative expression of mRNA
15
10
5
0
(Let) − + + +
E10arom LR3 LR9
PXR
Relative expression of mRNA
2.5
2
1.5
1
0.5
0
(Let) − + + +
E10arom LR3 LR9
