# Supplementary material for: Contribution of Estrone Sulfate to Cell Proliferation in Aromatase Inhibitor (AI) -Resistant, Hormone Receptor-Positive Breast Cancer
Source: PLoS One. 2016 May 26;11(5):e0155844. doi: 10.1371/journal.pone.0155844 (PMC4882040; doi:10.1371/journal.pone.0155844)

S3 Figure. Proliferation assay of LR cell lines and E10arom. A) Treatment of estrone (E1). **B)** Treatment of estradiol (E2). The error bars show standard deviation.

A B

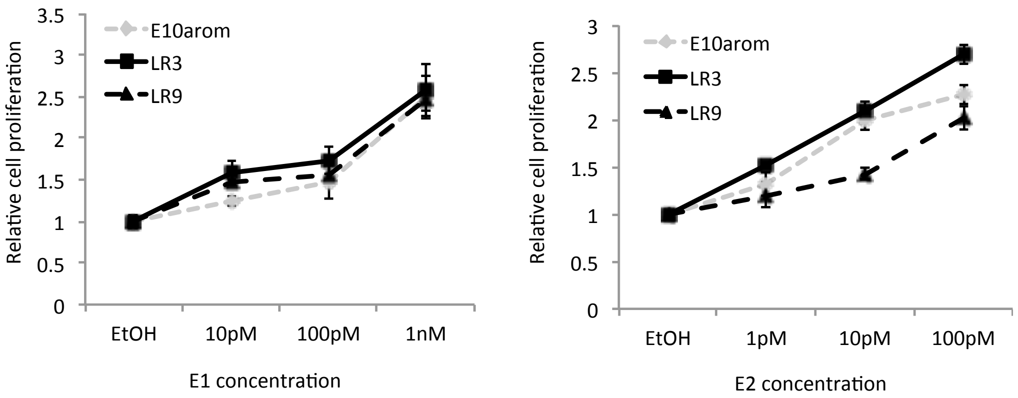

Supplement: S3 Fig — (PDF) [file pone.0155844.s003.pdf]
