# Supplementary material for: Contribution of Estrone Sulfate to Cell Proliferation in Aromatase Inhibitor (AI) -Resistant, Hormone Receptor-Positive Breast Cancer
Source: PLoS One. 2016 May 26;11(5):e0155844. doi: 10.1371/journal.pone.0155844 (PMC4882040; doi:10.1371/journal.pone.0155844)

S4 Figure. Relative estrogen receptor (ER) activity assay. Letrozole-resistant (LR) and parental E10arom cells treated with STX64. **A)** E1, **B)** E2. Error bars show standard deviation. ** $p < 0.01$.

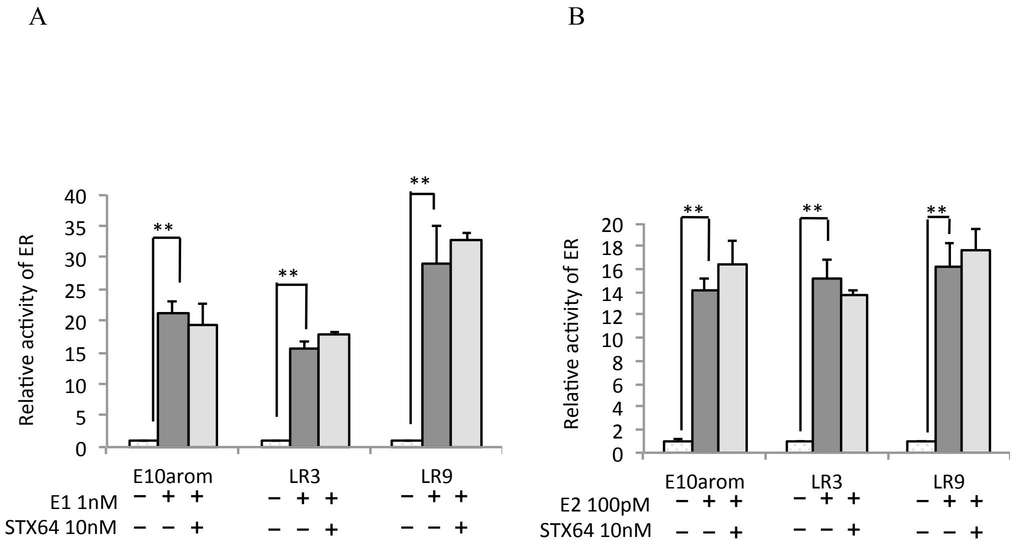

Supplement: S4 Fig — (PDF) [file pone.0155844.s004.pdf]
