# Supplementary material for: Contribution of Estrone Sulfate to Cell Proliferation in Aromatase Inhibitor (AI) -Resistant, Hormone Receptor-Positive Breast Cancer
Source: PLoS One. 2016 May 26;11(5):e0155844. doi: 10.1371/journal.pone.0155844 (PMC4882040; doi:10.1371/journal.pone.0155844)

S5 Figure. E1S-dependent cell growth in MCF-7 cells overexpressing STS. A) Vector map of STS overexpression model. To establish cell lines overexpressing steroid sulfatase (STS, cell line termed MCF-STS), the human STS gene in pRC/CMV was stably expressed in MCF-7 cells described in material and method section. **B)** Proliferation assay of treatment of E1S. gMCF-7 overexpressing STS cells were cultured in medium containing 5% DCC-FCS for three days and transferred to 24-well plates (10,000 cells/well) in medium containing 5% DCC-FCS and E1S. 5 days (MCF-STS) after addition of the test compounds and were counted using a micro cell counter.

A

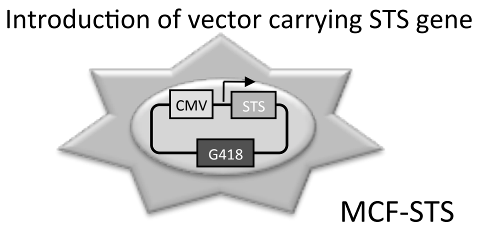

B

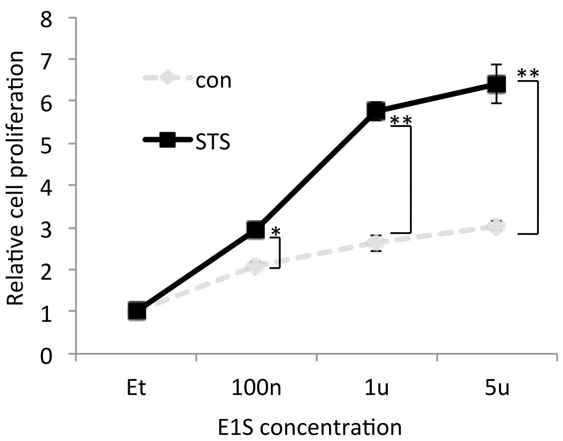

Supplement: S5 Fig — (PDF) [file pone.0155844.s005.pdf]
