# Supplementary material for: Contribution of Estrone Sulfate to Cell Proliferation in Aromatase Inhibitor (AI) -Resistant, Hormone Receptor-Positive Breast Cancer
Source: PLoS One. 2016 May 26;11(5):e0155844. doi: 10.1371/journal.pone.0155844 (PMC4882040; doi:10.1371/journal.pone.0155844)
Supplement: S6 Fig — (PDF) [file pone.0155844.s006.pdf]

S6 Figure. Effect of organic anion transporter peptide 4A1 (OATP4A1) siRNA on the proliferation of letrozole-resistant (LR) cells. A) Expression of OATP mRNAs in LR cells. Values were normalized to that of RPL13A but not converted to logarithms. **B)** siRNA-mediated inhibition of OATP4A1 mRNA expression. Control transfectants received scrambled siRNA. Values were normalized to that of RPL13A. **C)** Relative proliferation of LR cells expressing OATP4A1 siRNA. Transfection was performed according to the manufacturer's protocol. Cells received the indicated concentrations of estrone sulfate (E1S) 48 h after transfection and were harvested 96 h later. The vertical axis indicates cell number relative to vehicle-treated cells. Error bars show standard deviation. * $p < 0.05$. ** $p < 0.01$.

A

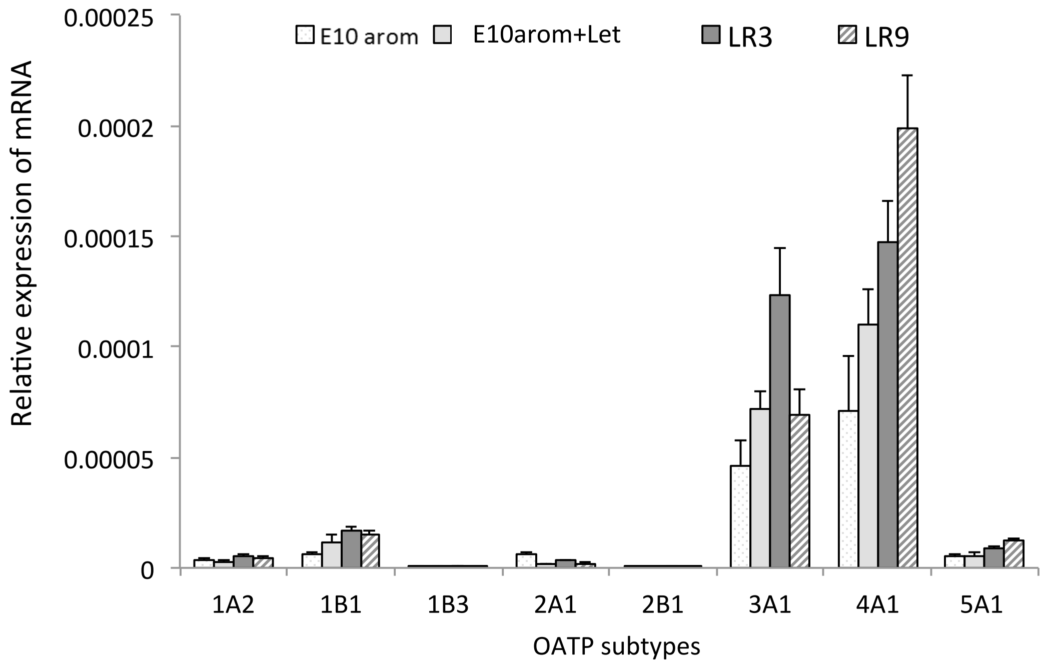

B

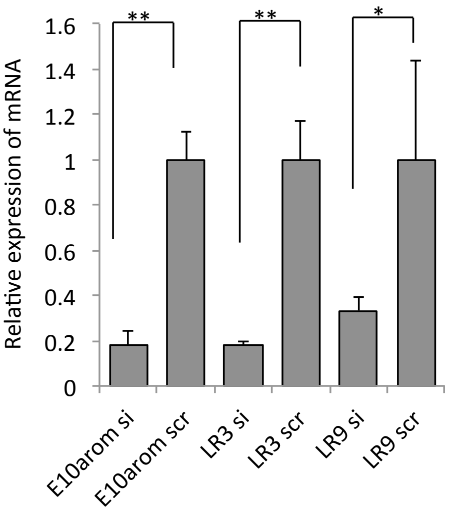

**
**
*
1.6
1.4
1.2
1
0.8
0.6
0.4
0.2
0
Relative expression of mRNA
E10arom si
E10arom scr
LR3 si
LR3 scr
LR9 si
LR9 scr

C

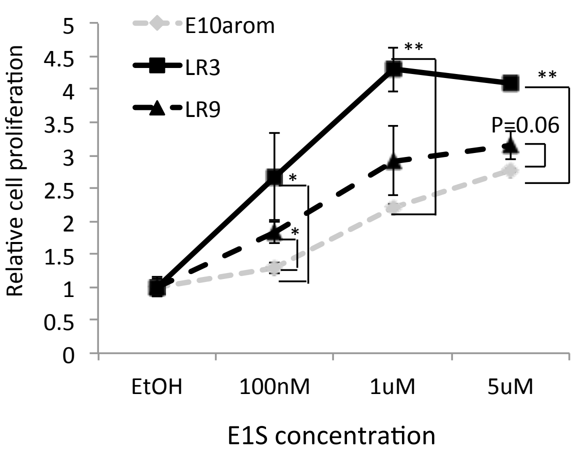

E10arom
LR3
LR9
5
4.5
4
3.5
3
2.5
2
1.5
1
0.5
0
Relative cell proliferation
**
**
P=0.06
*
*
EtOH
100nM
1uM
5uM
E1S concentration
