# Supplementary material for: Contribution of Estrone Sulfate to Cell Proliferation in Aromatase Inhibitor (AI) -Resistant, Hormone Receptor-Positive Breast Cancer
Source: PLoS One. 2016 May 26;11(5):e0155844. doi: 10.1371/journal.pone.0155844 (PMC4882040; doi:10.1371/journal.pone.0155844)
Supplement: S1 Table — (PDF) [file pone.0155844.s007.pdf]

S1 Table Primer sequences used in this study. The final concentration of the primers used for real-time polymerase chain reaction is shown in the column on the right. F forward primer, R reverse primer.

Primer data used in real-time PCR analysis.

Target mRNA	Sequence	(5') (3')	Final (nM)
ABCB-1	F	TGC CTT CAT CGA GTC ACT G	500
	R	TCT AAC AAG GGC ACG AGC TA	
AKR1C3	F	GCC TAG ACA GAA ATC TCC AC	500
(HSD17B5)	R	TCT GGT AGA CAT CAG GCA AA	
AR	F	ATG TGG AAG CTG CAA GGT CT	300
	R	CGA AGA CGA CAA GAT GGA CA	
Bcl-2	F	GTG GAT GAC TGA GTA CCT GAA C	300
	R	GCC AGG AGA AAT CAA ACA	
BRCP	F	GAT ATG GAT TTA CGG CTT TGC	500
	R	CCA AAT ATT CTT CGC CAG TAC A	
CAR	F	TTT CAT GGT ACT GCA AGT CAT C	500
	R	GGA GAC AGA AAG TGG TAT TGA G	
Cyclin D1	F	GGA GCC CGT GAA AAA GAG	500
	R	CAG GTT CCA CTT GAG CTT GT	
CYP3A4	F	AGT AAG GAA AGT AGT GAT GGC	500
	R	GCT GTT GAC CAT CAT AAA AG	
EST	F	TTG CCA CCT GAA CTT CTT CCT GCC	500
	R	TTG GAT GAC CAG CCA CCA TTA GAA	
ERα	F	CTC CCA CAT CAG GCA CAT	500
	R	CTC CAG CAG CAG GTC ATA	
HER2	F	GC TGA ACA ATA CCA CCC	500
	R	AGA CCC CTC CTT TCA AGA	
HSD3B1	F	GAA AGG TAC CCA GCT CCT GTT A	500
	R	ACA AGT GTA CAG GGT GCC G	

Table S1 continuing

Primer data used in real-time PCR analysis.

Target mRNA	Sequence	(5') (3')	Final (nM)
HSD17B1	F	GTG GAC GTG CTG GTG TGT A	500
	R	GAA AGG CAG CCC CAT CAA	
HSD17B2	F	GCG GCT GTG ACC ATG TTC T	500
	R	TGT CAC TGG TGC CTG CGA T	
KLK3	F	TGT CCG TGA CGT GGA TT	200
	R	ACG AGA GGC CAC AAG CA	
OATP1A2	F	ATG TGG ATT GCA ACT GTC CA	500
	R	TCA CAA CCA GCA AGA CAA GC	
OATP1B1	F	CAA ACT GAA CAC CGT TGG AA	500
	R	GTC CGG CAA CTG ATT TGT TT	
OATP1B3	F	AAG CAC TTG CAA TGG GTT TC	500
	R	AGC TGT TGG TGG ACC ACT TC	
OATP2A1	F	TGC GCC TAC TAT GAC AAC GA	500
	R	TTC TTC ACC CTC CAG CTG AT	
OATP2B1	F	GTT CCA TAA CAT CAA GCT GTT C	500
	R	TGC TCC ACC CCT TGT GT	
OATP3A1	F	CTA CCT GGT GAG CGT CCT GA	500
	R	CCC GAA GTA GCT CAC GAA GA	
OATP4A1	F	CAG AGA AAG CCC CTC CTT CT	500
	R	GGT CAC GGA CAC ATC GTA GA	
OATP5A1	F	GCC CAA CCA TCA GCT ATC AT	500
	R	ATG CAA GTG TTC GCA ACA AA	
PgR	F	AGC TCA CAG CGT TTC TAT CA	500
	R	CGG GAC TGG ATA AAT GTA TTC	
PXR	F	AGG AGC AAT TCG CCA TTA CT	300
	R	TAG CAA AGG GGT GTA TGT CC	
RPL13A	F	CCT GGA GGA GAA GAG GAA AG'	500
	R	TTG AGG ACC TCT GTG TAT TT '	
SRD5A1	F	CAA GGG GAG GCT TAT TTG AA'	500
	R	TCA TGA TGC TCT TTT GCT CTA C	
STS	F	ACT GCA ACG CCT ACT TAA ATG	500
	R	AGG GTC TGG GTG TGT CTG TC	
