# Supplementary material for: Contribution of Estrone Sulfate to Cell Proliferation in Aromatase Inhibitor (AI) -Resistant, Hormone Receptor-Positive Breast Cancer
Source: PLoS One. 2016 May 26;11(5):e0155844. doi: 10.1371/journal.pone.0155844 (PMC4882040; doi:10.1371/journal.pone.0155844)
Supplement: S2 Table — (PDF) [file pone.0155844.s008.pdf]

S2 Table Real-time polymerase chain reaction protocols and IDs of the primers and probe sets.

A Real-time PCR protocol for cell lines

Temperature	Time	Cycle	Process
95°C	3 min	1	denaturation
95°C	5 sec	1	denaturation
60°C	10 sec	40	annealing and extension
			generation of melt curve

B Real-time PCR protocol for breast tissue samples

Temperature	Time	Cycle	Process
50°C	2 min	1	holding
95°C	20 sec	1	holding
95°C	1 sec	40	denaturation
60°C	20 sec	40	annealing and extension

C IDs of the primers and probe sets for analysis of clinical samples

Gene	ID
STS	Hs00996676_m1
OATP1A2	Hs00366488_m1
OATP1B3	Hs00272374_m1
OATP4A1	Hs00983988_m1
OATP5A1	Hs00229597_m1
